# Cerebral palsy in children: subtypes, motor function and associated impairments in Addis Ababa, Ethiopia

**DOI:** 10.1186/s12887-021-03026-y

**Published:** 2021-12-03

**Authors:** Selamenesh Tsige, Ayalew Moges, Amha Mekasha, Workeabeba Abebe, Hans Forssberg

**Affiliations:** 1 Department of Pediatrics and Child Health, College of Health Sciences, Addis Ababa University, Addis Ababa, Ethiopia; 2 Department of Women’s and Children’s Health, Karolinska Institute and Astrid Lindgren Children’s Hospital, Stockholm, Sweden

**Keywords:** Cerebral palsy, Children, Subtypes, Impairments, Motor function, Ethiopia

## Abstract

**Background:**

Although, there is no population-level data in Ethiopia, a previous retrospective hospital-based study identified CP as the most common developmental disability in children. The overall aim of this study is to describe the clinical spectrum of CP in Tikur Anbessa Specialized Hospital in Addis Ababa, including CP subtype, gross and fine motor function, presence and pattern of associated impairments, and possible risk factors in children aged 2 to 18 years.

**Methods:**

A hospital-based descriptive cross-sectional study conducted- July – September of 2018 among 207 children with suspected motor symptoms. The Surveillance of CP in Europe (SCPE) decision tree was used as a guideline for inclusion and evaluation was by standardized questionnaire and clinical examination. Descriptive, bivariate and multivariate statistical analyses, Chi-square test, crudes association and adjusted odds ratio with 95% confidence interval employed.

**Result:**

One hundred seventy four children who fulfilled the clinical criteria were included. Half (50.6%) were under the age of 5 years with a mean age of 5.6 (SD 3.6) years; 55.2 were male. The majority had bilateral spastic CP (60.4%) followed by unilateral spastic CP 21.8%, dyskinetic CP 10.4%, and ataxic CP 3.4%; 4% were unclassifiable. Of the children, 95.4% had speech difficulty, 87.4% learning disabilities, 60.9% epilepsy, 24.7% visual impairment and 8.6% hearing impairment. On gross motor function (GMFCS) and manual ability (MACS) classification systems, 75.3% of the children had level IV and V functional impairment. More than 80% of the mothers had complications during delivery Half of the neonates did not cry immediately after birth,44% were resuscitated with bag mask ventilation at birth and 64% immediately admitted to NICU. During the first month of life, 50% had infection, 62% had trouble feeding, 49.4% had difficulty breathing, 35% had seizure and 13.8% had jaundice.

**Conclusion:**

The severe forms of CP predominate; most children are dependent on their parents for routine activities of daily living and cannot communicate well. Multidisciplinary care approaches and focused functional habilitation services are needed. Causal relationships cannot be drawn from these data but findings make a strong argument for improving maternal and child health care.

**Supplementary Information:**

The online version contains supplementary material available at 10.1186/s12887-021-03026-y.

## Background

Cerebral Palsy (CP) is one of the most common developmental disabilities in children worldwide and also in low- and middle-income countries (LMIC), however, there is a lack of robust population-based studies in Africa [1]. Until recently there were only studies on hospital clinical samples suggesting prevalence ranging from 2 to 10 cases per 1000 children from Egypt, Uganda, South Africa and South Egypt [2–5]. A few rigorous population-based studies have recently been published from Uganda [6] and Bangladesh [7] revealing large differences in prevalence from High Income Countries (HIC) [ 8]. The Ugandan and Bangladesh studies showed higher prevalence of CP of 2.9 and 3.4 per 1000 children respectively, compared to about 2.1 per 1000 in HIC. The etiological risk factors identified in Uganda were also very different from HIC, with almost no preterm born children, in contrast to 40%preterm born children in HIC, and numerous cases due to post neonatal infections (e.g., malaria). These studies clearly show that information cannot be generalized from studies in HIC and more studies on CP from LMIC are needed.

Andrews et al. (2019) showed that children with CP in Uganda lack access to health care, assistive devices, and education, which likely contributed poorer mobility and self-care skills [9]. A national survey on children with disabilities in Ethiopia in 2014 suggests that the vast majority of children with disabilities are not in school, and anecdotal evidence suggests many do not have access to community-based habilitation [10, 11].

However, CP was not specifically included in this study despite it being one of the most comprehensive disability surveys in Ethiopia. There is a significant lack of literature regarding the clinical and sociodemographic features of children with CP in Ethiopia. Even, the prevalence of CP is not known. The aim of this study was to describe clinical subtypes, motor and associated impairments and risk factors for CP using comprehensive methodology and terminology with a clinical cohort of children with CP at the University Hospital in Addis Ababa, the capital of Ethiopia.

## Methods

### Study setting

This was a prospective, hospital-based, descriptive cross-sectional study. Data was collected from July to September of 2018 at the outpatient Pediatrics Neurology Clinic (PNC) in Tikur Anbessa Specialized Hospital (TASH), Department of Pediatrics and Child Health, Addis Ababa. TASH is the largest teaching referral hospital in Ethiopia with over 700 beds. About 500–700 children visit the PNC every month. The monthly patient clinic registry of this clinic shows that, 25–30% are diagnosed with CP on follow up. Two thirds of patients visiting this clinic are from Addis Ababa, and the rest are referred from the countryside.

### Participants and procedures

A total of 174 children with confirmed diagnosis of CP, ranging in age from 2 to 18 years, were included in the study. The participants were recruited from 207 children with suspected motor symptoms. Of these children, 31 were new referrals to the PNC while 176 were children who had earlier visited the clinic and been diagnosed with CP by less stringent diagnostic procedures.

The assessments were conducted in three steps by the principal investigator (ST) and two general practitioners (GP). In the first step, ST screened all children in both groups using targeted history, physical examination and chart review. Children with obstructive hydrocephalus (*n* = 2), extra cerebral birth defects (n = 2), progressive motor disorder (*n* = 11), muscle hypotonia (*n* = 7) or presence of an isolated spinal neural tube defect (*n* = 3) were excluded. In total 25 children, 11 from the first group of 31 children and 14 from the 176 were excluded.

**Fig. 1:**
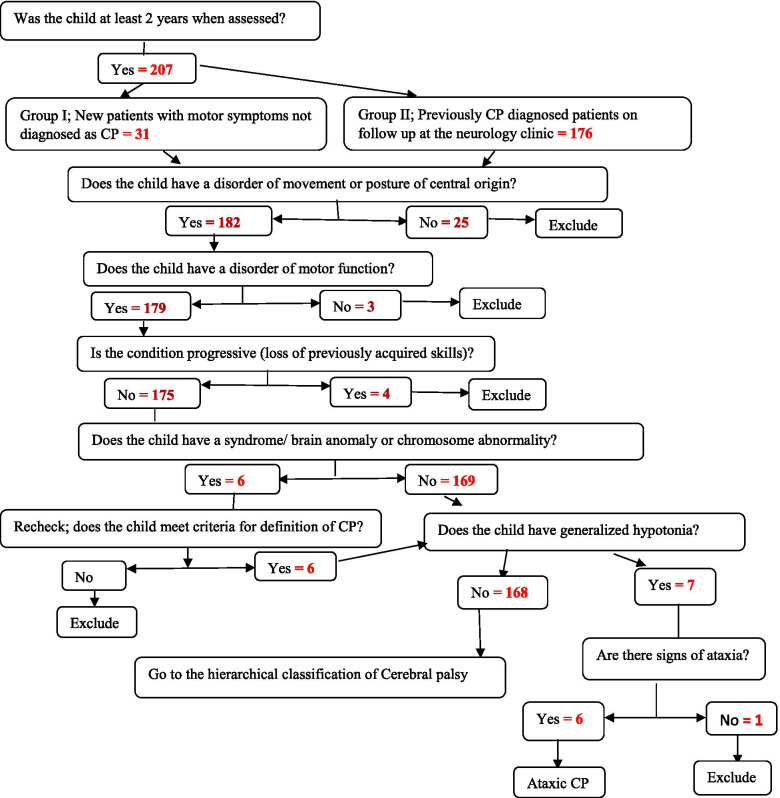
Steps undertaken on recruitment of children aged 2-18 years with confirmed diagnosis of CP based on the SCPE decision tree, Addis Ababa, Ethiopia, July – September 2018

In the second step, 182 children (20 from the first group and 162 from the second group) were examined by the GPs according to the SCPE decision tree [8]. All 20 children from the first group were confirmed to have CP, while 8 children from the second group, did not fulfill the criteria for CP. Among the eight who were excluded, three had only posture or movement abnormalities but no motor function abnormality, four had loss of previously acquired skills, and one had generalized muscle hypotonia without symptoms of ataxia. The procedures and steps to select study participants followed the SCPE decision tree [8] to select the study participants, as clearly outlined on Fig. 1.

In the third step, the 174 children with confirmed CP included in the study population were assessed by structured questionnaire and neurologic examination. .

A pre-tested and pre-coded questionnaire was used to interview caregivers and assess sociodemographics and information on prenatal, perinatal and postnatal risk factors. Information about associated impairments was also collected using a protocol based on the UNICEF/ Washington Group Child Functioning Module. These included intellectual disability, behavioral abnormalities, speech difficulties, feeding difficulties, visual impairment,hearing impairments and seizures.

Finally, a standard neurological examination to classify the CP subtype and functional assessment using Gross Motor Functional Classification System (GMFCS) and Manual Ability Classification System (MACS) was completed. Complete definition of variables and details of assessment is depicted on the Supplementary Information, section I.

### Quality control

The principal investigator checked daily for completeness of data collection and verified physical examination findings and classification of subtype of CP for each child.

### Statistical analyses

Analysis was done using the Statistical Package for Social Sciences (SPSS) version 21. Descriptive statistics with frequency tables were completed to show the socio demographic characteristics, antenatal, perinatal and postnatal complications, and proportions of CP subtypes, functional severity levels, and the proportion of children with associated impairments. Differences in proportions were evaluated using Chi-square statistics, and *p*-values < 0.05 were considered statistically significant and reported. Bivariate analyses were done to explore for crude associations between CP subtype and the child’s functional level, antenatal, perinatal and postnatal complications and associated impairments. Multivariate analysis with adjusted odds ratio (AOR) was completed for those significantly associated variables controlling confounding factors to identify true associations.

## Results

### Clinical subtypes and severity of gross and fine motor impairments

Bilateral spastic CP was the predominant subtype (60.4%) followed by unilateral spastic CP (21.8%), dyskinetic CP (10.4%), ataxic CP (3.4%) and unclassifiable CP (4%). Among those with bilateral spastic CP, 88 children (84%) had quadriplegic and 12(6.9%) had diplegic CP. Left sided involvement was predominant among children with unilateral spastic CP.

Table 1 shows the Distribution of CP Subtype.

**Table 1:** Distribution of CP subtype among children aged 2–18 years in Tikur Anbessa Specialized Hospital July – September 2018

		Frequency	Percent
Subtype			
**Spastic bilateral**	2 limbs	12	6.9
	3 limbs	5	2.9
	4 limbs	88	50.6
**Spastic unilateral**	Right	15	8.6
	Left	23	13.2
**Dyskinetic**	Dystonic	9	5.2
	Choreo-athetotic	9	5.2
**Ataxic**		6	3.4
**Unclassifiable**		7	4

Table 2 shows the distributions of GMFCS and MACS levels. A majority of children presented with severe impairments in both gross motor (75% at GMFCS level IV-V) and fine motor functions (75% at MACS level IV-V). Only 14% had the milder levels (I-II) of impairments.

**Table 2:** Distribution of Gross motor function and manual ability classification among children with CP aged 2–18 years in Tikur Anbessa Specialized Hospital July – September 2018

Variable	LEVEL	Frequency	Percent
GMFCS	I	8	4.6
	II	16	9.2
	III	19	10.9
	IV	27	15.5
	V	104	59.8
MACS	I	13	7.5
	II	11	6.3
	III	19	10.9
	IV	22	12.6
	V	109	62.6

*GMFCS* Gross Motor Functional Classification System, *MACS* Manual Ability Classification System

### Associated impairments

The distribution of associated impairments is shown in Table 3. Among the 174 children, 30 had two associated impairments and 144had three or more associated impairments. Speech difficulties (95%) and intellectual disability (87%) were most common. Seizures were present in 61% of the children, and of these 85% had been treated with an anticonvulsant, and 90% had been taken to the “holy water” (traditional healing spiritual water) at least one time. Seizures were also the most common cause of hospital admission. Visual and hearing impairments were less prevalent at 24.7 and 8.6%, respectively. All children with visual or hearing impairments were sent for a specialist evaluation. As some of the children did not attend the specialist’s evaluation, the final confirmed diagnosis of visual and hearing impairments became 20.7 and 4.6%. On bivariate analyses of factors which may predict the severity of impairment of gross motor functions, visual impairment, speech difficulties, income, mother’s education, father’s education and bilateral spastic CP showed statistically significant association with level IV-V GMFCS (*P* < 0.05). Speech difficulties (*P* = 0.007) and bilateral spastic CP (*P* = 0.001) showed the strongest correlation with severe GMFCS level. On multivariate analysis these variables remained statistically significant and appeared to independently predict the likelihood of severe motor function impairment (subtype of CP AOR = 3, 95% CI 1.103–8.768,*P* = 0.032 and the presence of speech difficulty, AOR = 11.5, 95% CI 1.283–103.542, *P* = 0.029). Children with speech difficulty were 11.5 times more likely to be in GMFCS level IV-V, as compared to those without speech difficult. Children with bilateral spastic CP were 3 times more likely to be in GMFCS level IV-V as compared to the other subtypes as shown in Table 4.

**Table 3:** Distribution of associated impairments among children with CP aged 2–18 years in Tikur Anbessa Specialized Hospital July – September 2018

Associated impairments	Frequency	Percent
Speaking difficulties	166	95.4
Intellectual disabilities	152	87.4
Behavioral abnormalities	44	25.2
Seizure	106	60.9
Feeding difficulties	104	59.8
Visual impairment	36	24.7
Hearing impairment	8	8.6

**Table 4:** Multivariate analysis of variables with the likely hood of severe motor function impairment (level IV-V) among children with CP aged 2–18 years in Tikur Anbessa Specialized Hospital July – September 2018

Language/speech difficulty	***P***-value		AOR	95% CI
	.029	11.527	1.283	103.542
**Visual**	**.083**	**.225**	**.042**	**1.215**
**Income**	**.910**	**1.080**	**.287**	**4.056**
**Mother education**	**.771**	**1.464**	**.113**	**19.014**
**Father education**	**.993**	**.991**	**.149**	**6.596**
**Bilateral spastic CP**	**.032**	**3.110**	**1.103**	**8.768**

On bivariate analysis of factors which may predict the severity of upper extremity impairment (MACS); visual impairment, speech difficulties, income, GMFCS and bilateral spastic CP showed statistically significant association with level IV-V MACS (*P* < 0.05). However, on multivariate analysis adjusting for potential confounders, only the presence of more severe impairment on gross motor functions remained statistically significant (AOR = 27.163, 95%CI: 9.902, 74.517, *p* value = 0.00) as shown in Table 5.

**Table 5:** Multivariate analysis of variables with severity of manual ability impairment among children with CP

Speech/language	P-value		AOR	95% CI
	.096	3.605	.797	16.308
**Visual**	.437	.626	.192	2.040
**Income**	.173	.498	.182	1.359
**GMFCS**	.000	27.163	9.902	74.517
**Subtype of CP**	.630	1.111	.725	1.702

### Antenatal and perinatal risk factors

Information about the antenatal, perinatal and postnatal period is presented in Tables 6 and 7. There were few remarkable events during antenatal care; in more than half of the mothers the duration of labor was less than 24 h. Notably, fewer than 10% of children were born preterm and none with birth weight below 1000 g. However 95% had complications during delivery identified as fetal distress (42.7%) followed by prolonged rupture of membrane (PROM) (25.9%).

**Table 6:** Identified antenatal factors among children with CP aged 2–18 years in Tikur Anbessa Specialized Hospital July – September 2018

	F		%
**Mother having ANC**	**Yes**	**151**	**86.8**
	**No**	**14**	**8**
	**DN**	**9**	**5.2**
**Maternal ingestion of alcohol during pregnancy**	**Yes**	**0**	**0**
	**No**	**165**	**94.8**
	**Unknown**	**9**	**5.2**
**Delivery place**	**Home**	**13**	**7.5**
	**Health institution**	**151**	**86.8**
	**Unknown**	**10**	**5.7**
**Gestational age**	**Term**	**136**	**78.2**
	**Pre-term**	**16**	**9.2**
	**Post term**	**12**	**6.9**
	**Unknown**	**10**	**5.7**

**Table 7:** Identified perinatal and postnatal factors among children with CP aged 2–18 years in Tikur Anbessa Specialized Hospital July – September 2018

	F		%
PROM	**Yes**	**45**	**25.9**
	**No**	**115**	**66.1**
	**Unknown**	**14**	**8**
Mode of delivery	**SVD**	**130**	**74.7**
	**Instrumental**	**16**	**9.2**
	**C/S**	**17**	**9.8**
	**Unknown**	**11**	**6.3**
Complications during delivery	**APH**	**4**	**2.5**
	**Birth trauma**	**3**	**1.9**
	**Neonatal RD**	**67**	**42.7**
	**HTN**	**7**	**4.5**
	**Precipitated labor**	**18**	**11.5**
	**Instrumental delivery**	**16**	**10.2**
	**Preterm**	**16**	**10.2**
	**C/S for big baby**	**3**	**1.9**
	**Post term**	**12**	**7.6**
	**Unknown**	**11**	**7**
Did the baby cry?	Yes	55	31.6
	No, but < 5 min	10	5.7
	No, > 5 min	81	46.6
	Unknown	28	16.1
Bag mask ventilation	Yes	77	44.3
	No	79	45.4
	Unknown	18	10.3
NICU admission	Yes	111	63.8
	No	63	36.2
Birth weight	1000–1500	6	3.4
	1500–2500	30	17.2
	2500–4000	83	47.7
	> 4000	3	1.7
	Unknown	52	29.9
Birth order	1st	102	58.6
	2nd	29	16.7
	3rd and above	35	20
	unknown	8	4.6

**Fig. 2:**
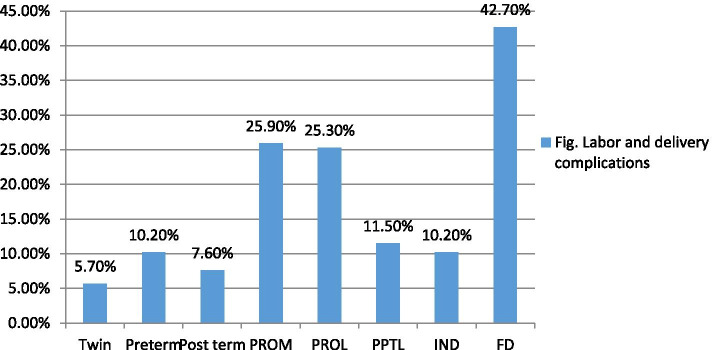
Distribution of Labor and delivery complications among children with CP aged 2 -18 years in Tikur Anbessa Specialized Hospital, July – September 2018

Figure 2 shows distribution of labor and delivery complications among children with CP. Almost two thirds of the children were admitted to the NICU; the most common causes of admission were respiratory distress (76.6%) and infection (11.7%). In the postnatal period, 54% had infection (4.2% meningitis and 3.2% tetanus), 62% had trouble feeding, 49.4% had difficulty breathing, 35% had seizure and 13.8% had jaundice with acute bilirubin encephalopathy.

Cross tabulation of perinatal factors showed that, the presence of fetal distress and PROM were associated with higher frequency of spastic CP subtypes. Precipitous labor was associated with higher frequency of dyskinetic or ataxic forms of CP. Neonates who required bag mask ventilation had higher prevalence of spastic CP. Neonatal Seizures were more common in children with spastic CP. Frequency cross tabulation is shown on Table 8.

**Table 8:** Cross tabulation of frequency distribution of perinatal factors with the subtype of CP aged 2–18 years in Tikur Anbessa Specialized Hospital July – September 2018

Labor/delivery complicatins *CP crosstabulation						
		CP subtypes				
			Spastic	Dyskinetic/Ataxic	Unclassified	Total
Labor/delivery complications	Fetal distress	Count	59	6	2	67
		% with in labor/ delivery complications	88.1	9.0	3.0	100
	Precipitated labor	Count	11	7	0	18
		% with in labor/ delivery complications	61.1	38.9	0.0	100
	Instrumental delivery	Count	13	1	1	15
		% with in labor/delivery complications	86.7	6.7	6.7	100
	Preterm delivery	Count	14	2	0	16
		% with in labor/delivery complications	87.5	12.5	0.0	100
	Others	Count	5	1	1	7
		% with in labor/delivery complications	71.4	14.3	14.3	100
	Unknown	Count	9	2	0	16
		% with in labor/delivery complications	81.8	18.2	0.0	100
	None	Count	31	5	3	39
		% with in labor/delivery complications	79.5	12.8	7.7	100

Table 9 shows a trend towards an association of jaundice with higher prevalence of dyskinetic / ataxic subtypes (OR = 2.663, 95% CI 0.946–7.497, *P* value = 0.064). The Pearson Chi square test also shows a similar trend (× 2 (1, *N* = 142) = 3.208, *p* value = 0.073). There was no association identified between the antenatal/perinatal factors and motor function impairment level.

**Table 9:** multivariate analysis of labor and delivery complications among children with CP aged 2–18 years in Tikur Anbessa Specialized Hospital July – September 2018

Labor & Delivery complications	Type of CP								
	Spastic		Dyskinetic/Ataxic		Unclassified		*P*	OR	95%CI
	F	%	F	%	F	%			
Fetal Distress	59	88.1	6	9	2	3	0.103	2.044	0.855–4.883
Preterm labor	11	61.1	7	38.9	–		0.014	2.512	1.265–4.988
Instrumental delivery	13	86.7	1	6.7	1	6.7	0.736	1.444	0.171–12.232
Post term delivery	14	87.5	2	12.5	–		0.688	1.556	0.185–13.108
PROM	41	91.1	2	4.4	2	4.4	0.047	1.151	1.011–1.310
Cried immediately after birth	Yes = 40	74.1	12	22.2	2	3.7		1.00	
	No = 102	85.7	12	10.1	5	4.2	0.071	1.815	00.967–3.408
Bagged after birth	Yes = 67	87	7	9.1	3	3.9	0.533	1.247	0.318–4.890
	No = 60	76.9	15	19.2	3	3.8			
Seizure	Yes = 53	86.9	7	11.5	1	1.6	0.452	1.430	0.563–3.635
	No = 89	79.5	17	15.2	6	5.4			
Jaundice	Yes = 16	69.6	7	30.4	–		0.064	2.663	0.946–7.497
	No = 126	84	17	11.3	7	4.7			

#### Socio demographic characteristics

Details of socio demographic characteristics are presented in Table 10. The mean age was 5.6 (SD 3.6) years with a slight male predominance. Seventy percent of the children above 5 years did not attend school; and among those who attended, one third performed on the last ten ranks in the respective classroom, and 20% had repeated a grade. Mothers alone were the primary care givers in 24.7% of the children. Both parents cared for 56.9% of children (mothers being the primary caregivers supported by fathers). The majority of the mothers (70.4%) were homemakers.

**Table 10:** Socio demographic characteristics of the children with CP aged 2–18 years in Tikur Anbessa Specialized Hospital July – September 2018

Socio demographic characteristics of the primary care givers		
Variable	Frequency	Percent
**Primary care giver**		
Mother alone	43	24.7
Father alone	18	10.3
Both parents but mother giving home care	99	56.9
Relative	4	2.3
Orphanage	10	5.7
**Marriage**		
Single (Never married)	11	6.3
Married currently	135	77.6
Single (Divorced)	12	6.9
Single (Widowed)	6	3.4
Unknown	10	5.7
**Mother education**		
Illiterate	20	14.1
Read and write	4	2.8
Primary	47	33.1
Secondary	39	27.5
College	32	22.5
**Father education**		
Illiterate	5	4.3
Read and write	2	1.7
Primary	26	22.2
Secondary	41	35
College	43	36.8
**Mothers occupation**		
Governmental	25	17.6
House wife	100	70.4
Merchant	3	2.1
Private	2	1.4
Daily laborer	12	8.5
**Fathers occupation**		
Governmental	49	41.9
Merchant	9	7.7
Private	27	23.1
Daily laborer	20	17.1
Farmer	6	5.1
Jobless	6	5.1
**Income**		
< 1250 birr(< poverty line)	22	12.6
**Average income = 3740 birr**		
>/= 1250 birr	124	71.3
Unknown	28	16.1

## Discussion

This clinical cohort of children with confirmed CP diagnosis from the main tertiary hospital in Ethiopia included a large cohort of children with severe motor impairments and seizures.. In most children, one or several perinatal risk factors were identified, revealing the potential to prevent the brain injury if properly treated. A majority of children did not attend school and were cared for by their mothers at home.

### Comparison with other clinical and population-based cohorts

The most recent population-based studies from LMIC were reported on children with CP in Uganda [6] and Bangladesh [7]. As expected,clinical findings differs in our sample likely due to more representative sample in those studies. The Bangladesh CP register involved 726 children with CP aged 4.8 months to 18 years; the majority (79.6%) had spastic CP similar to this study. The Ugandan study involved 31,756 children of which 442 had confirmed CP; spastic unilateral CP was the most common subtype (46%) followed by bilateral CP, which is reversed in case of our study. Major differences between our studies were seen in the GMFCS and MACS level reported. Two thirds of the children in our cohort had a level IV-V versus less than 50% of the children in the Ugandan and Bangledesh cohort.

The difference in distribution between these population-based studies and our study versus HIC is presented in the Supplementary Table.

The clinical panorama in this study is however similar to other hospital based descriptive studies in LMIC including Uganda, Egypt, Cameroon, Botswana [4, 5, 12–14] and North India [15, 16]. These studies were in referral and university affiliated hospitals involving children with CP below 18 years of age using similar diagnostic criteria to our study. Bilateral spastic CP was the predominant subtype; 45% in Molago hospital of Uganda, 72% in Egypt, 26.7% in Yaounde vs 50.6% in our study. Cognitive impairment and epilepsy were the most common associated impairments identified in these studies as well. The rate of Cognitive impairment was 91% in India, 75% in Uganda, 77% in Egypt, 84% in Gaborone referral hospital and 40% in Younde vs 87.4% in our study. Sever motor function impairment as per the GMFCS and MACS was also reported; 37% in Uganda and 41% in Botswana referral hospital vs 75% in our study. Similar to our study, associated impairments were most frequent in children with spastic and dyskinetic cerebral palsy in these clinical cohorts.

The rate of speech difficulty in our cohort differed largely from the other African studies outlined above; however, it is comparable to a descriptive study done in a rehabilitation referral center in India [16] which reported 83.7%. The high rate was found to be associated with the severity of motor function in our study.

The high numbers of children with severe (quadriplegic) bilateral spastic CP suggest injuries to the full term brain during the birth process (hypoxic ischemic injury) or acquired infections such as meningitis or encephalitis [4]. Mild bilateral spastic CP is often seen in the preterm brain and may reflect that the Ethiopian system is not able to support preterm neonates for long-term survival [17]. Our findings regarding the amount of preterm CP patients were similar to the findings in Uganda [6] and unlike the Australian study where 43% of children with CP were born preterm [18].

We identified that children with spastic subtypes of CP had higher rates of fetal distress and PROM and were later found to have higher rates of language difficulty and more significant functional impairments than the other CP subtypes. Those with dyskinetic and ataxic CP were found to have higher rate of precipitous labor and jaundice during the neonatal period.

This suggests that the spastic subtype of CP is likely associated with perinatal hypoxia as indicated above while dyskinetic and ataxic forms may be associated with bleeding and injuries to the deep grey matters of the brain that can occur in cases of precipitous labor [17]. However, because the possible causes were identified based on history given by the parents (no registries or MRI results were available) causal relationship of definitive risk factors cannot be determined based on this study.

By applying stricter criteria and procedures for determining the diagnosis of CP, we found that 22 of the 176 children who had been given the diagnosis at an earlier visit to the clinic, did not fulfill the SCPE criteria. We also changed the subtype of 20 children following comprehensives evaluation. Over the years, diagnostic criteria for CP has developed and the newer definition [19] is now used in most HIC. In combination with functional classifications systems for gross and fine motor, and communication, these approaches provide a comprehensive description of the child’s diagnosis, functional status and needs, which is very useful in clinical practice for planning interventions and for predicting prognosis. Older diagnostic systems were previously used in Ethiopia, and probably in many countries in sub-Saharan Africa and other LMIC. This impacted findings, for example a previous retrospective study from our hospital TASH showed 48.2% of unclassified CP [20], which differs significantly from the present study. Prospective assessment of children in this study provided high-quality systematic clinical information. Hopefully, this study, and studies from other countries in sub-Saharan Africa, can pave the way for this contemporary method to provide diagnostic and functional descriptions, which should also lead to improved clinical practice.

### Strengths and limitations

Strengths of this study include a prospective design and use of the contemporary international systems for CP diagnosis and motor function level assessment making it possible to compare with other cohorts. The sample was also large enough to perform some statistical analyses; however, causal relationships cannot be determined from our cross sectional study. We took a cut-off age of 2 years to include children with CP while the SCPE recommends age 5 years [8]. While a CP subtype is not confirmed until age 5 years as per the SCPE, the age of 2 years was chosen in this study as to best represent the patients being diagnosed with CP in the clinical environment of Ethiopia. It is also evident that early diagnosis is very important even if topography and severity estimates may not yet be possible or fully accurate.

There is a possibility that the sample may overestimate CP severity, since we completed the study at Tikur Anbessa Specialized Hospital, a hospital more prone to receive severe cases as the final level of referral in the setting. Additionally, it is a common practice for children with disabilities to not seek care due to fear of stigma, and parents thus may only bring them to medical attention in the face of significant burden.

Although this study cannot be generalized to the population level, we believe the fact that half of the participants presented at an early age (i.e. below 5 years of age) suggests the sample is more representative of the community.

Another limitation was that much of the information on risk factors and associated impairments was based on interviews and surveys of the caregivers which could have led to recall bias influencing our results in terms of false positive risk factors, exaggeration or underestimation of associated impairments.

## Conclusion

Most of the children in our study had severe functional impairments and were dependent on their caregivers for their daily living. This is a hard burden on the caregivers especially as there is poor access to rehabilitation services in Ethiopia. Proper diagnosis, including categorization of clinical subtypes, assessment of motor function and identification of co-morbidities is essential for early intervention and follow-up. The study also found that many children had adverse events during the perinatal period, many of which are prevented in HICs. This indicates the importance of further improvement in maternal and neonatal care in this country. One suggestion moving forward would be for early intervention of labour related complications and better antenatal monitoring. Unfortunately, our current system has significant limitations in this domain.

Increasing healthcare provider awareness could allow for early intervention and hopefully result in less perinatal brain injury and improved outcomes for patients. However, in addition to focusing on prevention, further changes need to be made in our long-term management of children with CP. The creation of multidisciplinary teams to manage these children will allow for comprehensive care of their social, medical and rehabilitation needs and this could be constructed following the ICF frame work [21].

## Supplementary Information

**Additional file 1.**

## Data Availability

The data sets used and/ analyzed during this study are available from the corresponding author on reasonable request.

## Abbreviations

AAU: Addis Ababa University
CHS: College of Health Science
CP: Cerebral Palsy
GDD: Global Developmental Delay
GMFC: Gross Motor Functional Classification
HIC: High Income Countries
ICF: International Classification of Functioning, Disability and Health
LMIC: Low and Middle Income Countries
PROM: Premature Rupture Of Membrane
PNC: Pediatrics Neurology Clinic
SCPE: Surveillance of Cerebral Palsy in Europe
TASH: Tikur Anbessa Specialized Hospital
