# Supplementary material for: Cerebral palsy in children: subtypes, motor function and associated impairments in Addis Ababa, Ethiopia
Source: BMC Pediatr. 2021 Dec 3;21:544. doi: 10.1186/s12887-021-03026-y (PMC8641229; doi:10.1186/s12887-021-03026-y)
Supplement: Supplementary file 1 — Additional file 1. [file 12887_2021_3026_MOESM1_ESM.pdf]

CEREBRAL PALSY IN CHILDREN: SUBTYPES, MOTOR FUNCTION AND ASSOCIATED IMPAIRMENTS; ADDIS ABABA, ETHIOPIA

Selamenesh Tsige (1), Ayalew Moges (1), Amha Mekasha (1), Workeabeba Abebe (1), Hans Forssberg (2)

1. *Department of Pediatrics and Child health, College of Health Sciences, Addis Ababa University*
2. *Department of Women's and Children's Health, Karolinska Institutet and Astrid Lindgren Children's Hospital, Stockholm, Sweden*

Supplement Information

I. Definition of variables

1. Socio demographic

The data collectors used a pretested and pre-coded questionnaire to interview the caregivers. Questions include the child's age, sex, level of education, medical history from pregnancy to the present, family history, the child's developmental milestones, past and present nutritional history, parental educational status, living condition and family history of illness.

2. General Clinical Examination

This included a standard neurological examination. The principal investigator ST crosschecked the examinations done and the Pediatric Neurologist (AM) was consulted for difficult cases.

3. Functional Assessment

The GMFCS and MACS were used to classify the gross motor and fine motor function of the children. These classification systems grade the motor impairments from more to less functional in a 5-level ordinal scale, where Level I indicates minor limitations and V extensive impairments.

4. Associated Impairments

The associated impairments were investigated using a structured questionnaire with the caregivers and chart review as per the UNICEF/ Washington group child functioning module. The impairments assessed are:

a. Intellectual disability:

We asked Care givers if the child has had any difficulty in understanding simple conversations, is unable to learn things that other children of the same age could and if the child appeared mentally slow compared to his peers.

b. Behavioral abnormalities:

We asked care givers if the child had any kind of abnormal or uncontrollable behavior and if a psychiatrist had evaluated the child previously. We documented the specific diagnosis for those children who previously had a psychiatry evaluation. .

c. Speech difficulties:

We asked the caregivers if the child has age appropriate speech using the language he understands.

d. Feeding difficulties:

We asked caregivers if the child had age appropriate feeding ability. We asked for swallowing difficulties, regurgitation of feeds, tongue thrust, tonic bite, chewing difficulties, vomiting of feeds or drooling saliva.

e. Seizures;

Seizure was defined as two or more afebrile seizures in the last 5 years that were spaced 24 hours apart and unrelated to acute infection, metabolic disturbance, or drugs.

f. Visual impairment:

We asked caregivers if they had any concern with the child's vision. We examined movement of the eyes following light or bright objects. We referred children with suspected visual impairment to Ophthalmology.

g. Hearing impairment:

We asked caregivers if they had any concern with the child's hearing. We examined response to sound. We referred children with suspected hearing impairment to ENT.

5. Risk factors

We asked caregivers specific questions with respect to Prenatal, perinatal and postnatal risk factors. Questions included a history of maternal infections, drug intake during pregnancy, gestational age, place of delivery, birth weight, complications during delivery, neonatal resuscitation, neonatal admissions and medical history during the first two years of life.

II. **Comparison Table of functional level and associated Impairments in Uganda, Bangladesh and Ethiopia Vs HIC**

GMFCS level	Uganda		Bangladesh	Ethiopia	HICs
	2–5 years	6–17 years			
I–II	39%	72%	32%	13.8 %	65%[1]
III	24%	18%	22%	10.9 %	3%[1]
IV–V	36%	10%	47%	**75.3 %**	33%[1]
**MACS level**					
I–II	48%	77%	39%	13.8 %	59%[1]
III	3%	13%	18%	10.9 %	9%[1]
IV–V	48%	10%	44%	**75.2 %**	32%[1]
**Intellectual disability**			39%		
Confirmed	45%	58%			33–45%[1,2]
Unconfirmed	24%	13%		87.4 %	26%[2]
**Seizures**			23%		28–44%[1-3]
Confirmed	30%	38%			
Unconfirmed	15%	17%		60.9 %	
**Speech**					
Non-verbal	45%	37%	35%	95.4 %	49%[4]
**Hearing impairment**			10%		12%[2]
Confirmed	3%	10%			
Unconfirmed	18%	15%		8.6 %	
**Vision impairment**			10%		17–35%[2]
Confirmed	15%	10%			
Unconfirmed	18%	23%		24.7 %	

III. Data Collection Tools

I; selection of cases to be included on the study

Code number ______________ Date ____/___/___

Please use the following SCPE decision tree to decide on inclusion of the child to the study NB; if the child was already diagnosed with CP but excluded according to this decision tree please notify the consultant neurologist and the primary investigator.

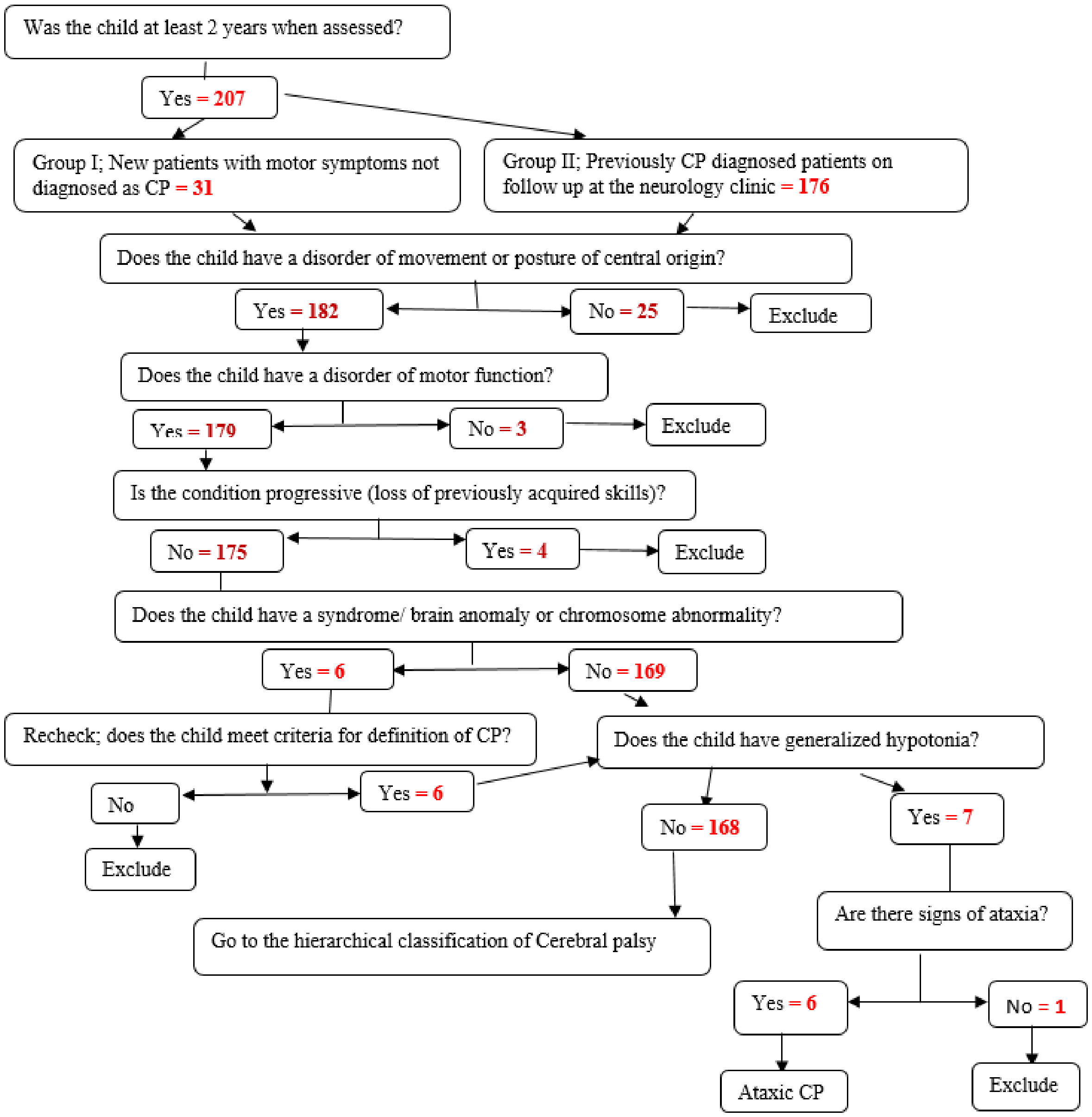

Go to the next page for children included on the study based on the decision tree

consent

የጥናቱ የመረጃ እና ስምምነት ሰነድ

ይህ የመረጃ እና የስምምነት ሰነድ በጥቁር አንበሳ ሆስፒታል የሚታከሙ በእርግዝና ወቅት ወይም በወሊድ ጊዜ በተከሰተ የአእምሮ ጉዳት ምክንያት ሰውነታቸውን የማዘዝ ችግር ያለባቸው ህፃናት ላይ ያለውን የጉዳት አይነት፤ የእንቅስቃሴ አቅማቸውንና በተጓዳኝነት የሚከሰቱ ችግሮችን በተመለከተ ለሚደረገው ጥናት የተዘጋጀ ነው።

<u>የጥናቱ መነሻ ሐሳብ እና አላማ</u>

የጥናቱ አላማ በእርግዝና ወቅት ወይም በወሊድ ጊዜ በተከሰተ የአእምሮ ጉዳት ምክንያት ሰውነታቸውን የማዘዝ ችግር ያለባቸው ህፃናት ላይ ያለውን የጉዳት አይነት፤ የእንቅስቃሴ አቅማቸውንና በተጓዳኝነት የሚከሰቱ ችግሮችን በወቅቱ በመለየት አስፈላጊው ህክምና እና ክትትል እንዲረግላቸው ለማድረግ ነዉ።

<u>ከእርሶ ምን ይጠበቃል</u>

ልጁ/ልጅቱ በጥናቱ እንዲሳተፉ ከፈቀዱ ስለ የማህበራዊ እና ጤና ሁኔታ መረጃ ይሰጣሉ።

<u>በጥናቱ ላይ በመሳተፍየሚያጋጥሙ ስጋቶች</u>

ልጅዎ በዚህ ጥናት በመሳተፉ የሚደርስበት አንዳችም ጉዳት አይኖርም። በጥናቱ ጊዜ ልጅዎ ያልታከመ የጎንዮሽ ጉዳት የተገኘበት እንደሆነ በአስቸኳይ አስፈላጊውን ህክምና በሚመለከተው ባለሙያ እንዲያገኝ ይደረጋል።

<u>ሚስጥር ስለመጠበቅ</u>

የጥናቱ የተሳታፊዎችን መረጃም ሆነ ማንነት በሚስጥር የሚጠበቅ ይሆናል በመሆኑም የተሳተፊዉ ስም በጥናቱ መጠይቅ ላይ አይካተትም።

<u>በጥናቱ ለመሳተፍ ስለአለመፈለግ ወይም ተሳትፎን ስለማቋረጥ</u>

በጥናቱ እንዲሳተፉ አይገደዱም እንዲሁም ተሳትፎዎን በማንኛውም ጊዜ ማቋረጥ ይችላሉ። በመሳተፎ ራስዎን ወይም ልጅዎን በተመለከተ መግለፅ የማይፈልጉት መረጃ ካለ እንዲገልፁ አይገደዱም። በጥናቱ መሳተፍ ባይፈልጉ በልጅዎ የህክምና ክትትል ላይ የሚያሳድረው ምንም አይነት ተጽእኖ አይኖርም።

<u>የጥናቱ ጥቅም</u>

ከዚህ ጥናት የሚገኘው መረጃ በጥቁር አንበሳ ሆስፒታል የሚታከሙ በእርግዝና ወቅት ወይም በወሊድ ጊዜ በተከሰተ የአእምሮ ጉዳት ምክንያት ሰውነታቸውን የማዘዝ ችግር ያለባቸው ህፃናት ላይ የሚደረገውን ህክምና እና ክትትል ለማሻሻል ይጠቅማል።

<u>በጥናቱ ወቅት ጥያቄ ቢኖሮት</u>

ማንኛውም ጥያቄ ካሎት ከዚህ በታች በተገለፀው የዋና ተመራማሪ አድራሻ በመጠቀም መጠየቅ ይችላሉ።

የስምምነትሰነድ

ልጁ/ልጅቱ በዚህ ጥናት እንዲሳተፍ(እንድትሳተፍ) ፍቃደኛ ስሆኑ ለትብብርዎ በቅድሚያ እያመሰገንን ከበታች በተዘጋጀው ቦታ ላይ እንዲፈርሙ በትህትና እንጠይቃለን።

ከዚህ በታች ስምና ፊርማዬ የተገለፀው ግለሰብ ከላይ የተገለፁትን መረጃዎች በማንበብ አና በመረዳት የጥናቱ ተሳታፊ ለመሆን ተስማምቻለሁ።

ስም:______________________________

ፊርማ:____________________________

ቀን:_____________________________

ዋና ተመራማሪ ፡ ዶ/ር ሰላሜነሽ ፅጌ

ስልክ: +251910101401

ኢሜል: selidoc2@gmail.com

III. Study Questionnaire

Title of the Research – Cerebral Palsy in children aged 2 – 18 years attending follow up at the Pediatric Neurology Clinic; clinical subtypes, motor function and co-morbidities

Instructions – Please take your time to complete the information below.

Code number _______________ Date ____/___/___

Part I – Socio-demographic Data of child

1. Age in completed years and months |___|___|
2. Sex;__________
3. Birth order: _______________
4. Family size |___|___|
5. Address (region)____________________
6. Religion:___________________
7. Child's level of education
   a. . No schooling b. Nursery c. Primary School (grades 1-8) d. Secondary school
8. Has the child ever repeated grade?___________
9. What is the child's average performance score in class
   a. Top ten b. 1st quarter c. 2nd quarter d. 3rd quarter e. 4th quarter f. Last ten

Part II–Family and Primary caregivers' information

1. Who is the primary caregiver? If not parents, state why____
   ____

a. Mother b. Father c. Both parents d. Adult relative e. Non relative adult f.Orphanage

2. What is the marital status of parents?
   a. Single parent b. Married c. Divorced d. Widowed (specify the deceased) ____________

3. Primary caregiver's highest level of education –**Mother**_____, **Father**_______, **Other**_____
   a. Illiterate b. Can read and write c. Primary (attended grades 1-8) d. Secondary (attended grades 9-12) e. College(certificate/diploma/1st degree/Master/MD/PhD)
4. Primary caregiver's occupation (**Mother**) _______________________(**Father)**__________________ (**Other**)___________________ and Monthly income_____________
5. Monthly income (as perceived by primary care giver to support the family):
1. Partially Adequate income 2. Inadequate income
6. Number of child death in home if any? If yes, what was the age and cause of death?
   a. Age _____ suspected cause of death____
   b. Age _____ suspected cause of death____
   c. Age _____ suspected cause of death____

Part III. Medical history

1. At the time of child conception/birth, was mother taking alcohol?
   a. Yes b. no c. don't know
2. If yes to no 7, specify years of taking alcohol_____________ . Type of alcohol_________________________. Volume of alcohol____________________________
3. When the mother was pregnant with the child, did she have ANC follow up?
   a. Yes b. no c. don't know
4. Any complications during ANC ___________________________________
5. Where was the child born?

a. Home b. Hospital c. Health center d. Other, specify___________________

6. Was it a single birth?

   a. Single birth b. Twins c. Triplets d. More

7. Was the baby born at term (between 37-42 weeks or at about 9 months)?

   a. Yes b. No, >3 weeks early c. No, > 2 weeks late

8. Was there prolonged rupture of membranes (>18 hours) before delivery?

   a. Yes b. no c. Don't know

9. How long was the labour?

   a. <24 hours b. >24 hours c. No labor, C-Section d. Unknown

10. Was there any complication during labor and delivery?

    ____

11. Did the baby cry immediately after birth?

    a. Yes b. No, but in <5 min c. No, after>5 minutes d. Unknown

12. Was the baby resuscitated with bag and mask at birth?______________________________

13. Was the baby admitted to hospital care at birth (NICU). If yes, specify why

    ____

14. What was the birth weight? ________________

15. Did the child have any of these conditions from birth to one month of age?

	**Condition**	**Response**
1	Seizures	
2	Infection	
3	Trouble feeding	
4	Meningitis	
5	Deep jaundice	
6	Severe diarrhea	
7	Difficult breathing	
8	Tetanus	

16. Growth and development:

a. Compared with his/her peers did your child have any difficulty or delay while growing up?
b. Delay in sitting?
c. Delay in standing?
d. Delay in walking?
e. Difficulty in walking/ moving limbs?
f. Difficulty in seeing during day or night?
g. Difficulty in hearing?
h. Unable to speak/ make appropriate sounds for age?
i. Difficulty in understanding what he/she is told?
j. Unable to learn things other age mates can?
k. Appears mentally slow compared to other children his/her age?
l. Other problem?, describe…………………………………………………….

17. Immunization History:

a. Is immunization completed? ______________________________
b. If no, what doses are missing?____

16. Was the child breast fed exclusively the 1st 6 months ?

a. yes b. No, specify what was initiated________________________________

17. At what age in months was solid food introduced___________ , how often is the child fed within 24 hours______________________________________

18. Can the child feed himself or herself?

a. Yes, skillfully b. No, must be fed c. Yes, but unskilled (i.e. like a baby) d.unknown

19. At what age did child start to finger feed himself or herself?

a. By 7mo b. By 1 year c. After 1 year d. After 2 years e. Not yet f. Unknown

20. If child has a feeding problem, which one (s)

a. Swallowing difficulties b. Regurgitation of feeds c. Tongue thrust d. Tonic bite e. Chewing difficulties f. Vomiting of feeds g. Drooling saliva=6

21. Has the child ever been hospitalized ?_____________

If yes, what was the reason for the most recent **3** admissions? Please describe:

(i)____

___

(ii)____

___

(iii)____

___

22. Is the child taking any medication? ____________

If YES, which drugs? Why

?____

23. Is there any alternative/traditional medicine this child is taking?_______If Yes,specify____

______ 24. Has the child ever been diagnosed with the following conditions;

	**CONDITION**	**RESPONSE**
**1**	Epilepsy?	
**2**	Attention Deficit Hyperactivity disorder?	
**3**	Hearing Impairment?	
**4**	Visual Impairment?	
**5**	Autism?	
**6**	Speech and Language impairment?	
**7**	Behavioural impairment?	
**8**	Mental illness?	
**9**	Cerebral Palsy?	

25. Are there any other illnesses that have been diagnosed in NAME's family members?_________

If yes (**specify illness**)____

Thank you!!

IV. CEREBRAL PALSY CLINICIAN'S ASSESSMENT QUESTIONNAIRE

Please carefully use the following assessment steps (adapted from SCPE) together with a quick neurological examination to decide whether the child has CP.

Code no______________________________ Date ___/____/________

NB; We are using the SCPE consensus definition of cp which consists the following 5 elements to define CP. Check this elements as you proceed with your clinical assessment to diagnose CP. According to SCPE, in order to define a disorder as CP, it should consist of the following five key elements:

1. It is permanent but not unchanging;
2. It involves a disorder of movement and/or posture
3. It involves a disorder of motor function;
4. It is due to a non-progressive interference/lesion/abnormality;
5. This interference/lesion/abnormality is in the developing/immature brain

Anthropometry

1. Weight _ _ Kg , W/A____________________________
2. Height/Length _ _ _ cm , H/A__________________________________
3. Occipital-frontal head circumference _ _ cm, Hc/A_______________________________
4. Mid Upper Arm Circumference (MUAC) _ _ cm,_______________________________
5. W/H____
6. BMI____

Clinical examination

Vitals (i) Pulse rate……… (ii) Temp…………… (iii) Respiratory rate…………

Gross and fine motor classification; see at the end of the questionnaire

GMFCS – E & R ;

GMFCS – E & R	MACS
Level I	MACS ; Level I
Level II	Level II
Level III	Level III
Level IV	Level IV
Level V	Level V

A. Observe the child in any comfortable position he/she may adopt and **check for abnormal involuntary movements**.**If present place a✓ (Tick) and if absent place a × (Cross) as appropriate.**

	**At rest**	**With excitement or goal-directed movement**
1.None		
2.Short jerky		
3.Slow and writhing (chorea)		
4.Tremor		
5.Flexor and extensor spasms		
6.Other please describe (tics, myoclonus, dystonia)		

B. Examine **muscle tone** in each limb and ✓**<u>tick one box</u>** for each limb. Test **tendon reflexes** in each limb and ✓**<u>tick one box</u>** for each limb.

	**Right Upper limb**	**Left Upper limb**	**Right Lower Limb**	**Left Lower limb**
1.Normal tone				
2.Increased tone				
3.Decreased tone				
4.Varying increased/decreased				
5.Normal tendon reflexes				
6.Increased tendon reflexes				
7.Decreased tendon reflexes				

C. **Test** for **clonus** and look for **contractures** and investigate **muscle size/volume**. (If present place a ✓**<u>tick one box</u>** for each limb if absent in any limb place a **<u>× in the box.</u>**

	**Right Upper limb**	**Left Upper limb**	**Right Lower Limb**	**Left Lower limb**
1.Clonus present				
2.Contractures present				
3.Muscle atrophy present				

D.Gait and posture pattern. Place a ✓ tick in the appropriate boxes

1.No gait problem, walks fluently	
2.Gait functional but non-fluent	
3.Abnormal gait reducing mobility; crouched gait	
4.Asymmetric gait	
5.Walking aids regularly used	
6.No independent walking	
7.Can sit independently	
8.Can sit only with support	
9.Cannot sit	

E.Upper Limb Function. Place a ✓ tick in the appropriate boxes

	**Right Upper Limb**	**Left Upper Limb**
1. Moves limb well. No apparent problem		
2.Can perform finger-nose according to age		
3.Can perform alternating hand movements		
4.Can perform “piano-playing-in-air”		
5.Can reach and grasp objects according to age		
6.Prefers using either the right or left hand		
7.Able to eat self with either hand		
8.Can use both hands at same time (bimanual skills)		
9.Can put on vest or T-shirt without help		
10.Physically incapable of putting on vest or T-shirt and unable to feed self with either hand		

F. Distribution of **Symmetric/Asymmetric Limb involvement**:✓Tick as appropriate.

	**YES**	**NO**
1. Is there Right to Left asymmetry of tone or function?		
2. If yes; is right side worse?		
3. If yes; is left side worse?		
4. Are the upper limbs more affected than the lower limbs?		

5. Are the lower limbs more affected than the upper limbs?		

G. **Making a Cerebral Palsy diagnosis:**

Does this child have Cerebral Palsy? If Yes or Uncertain - proceed with **H** and subsequent examinations. If No - proceed with **I**.

H.If **YES**, use the classification tree below

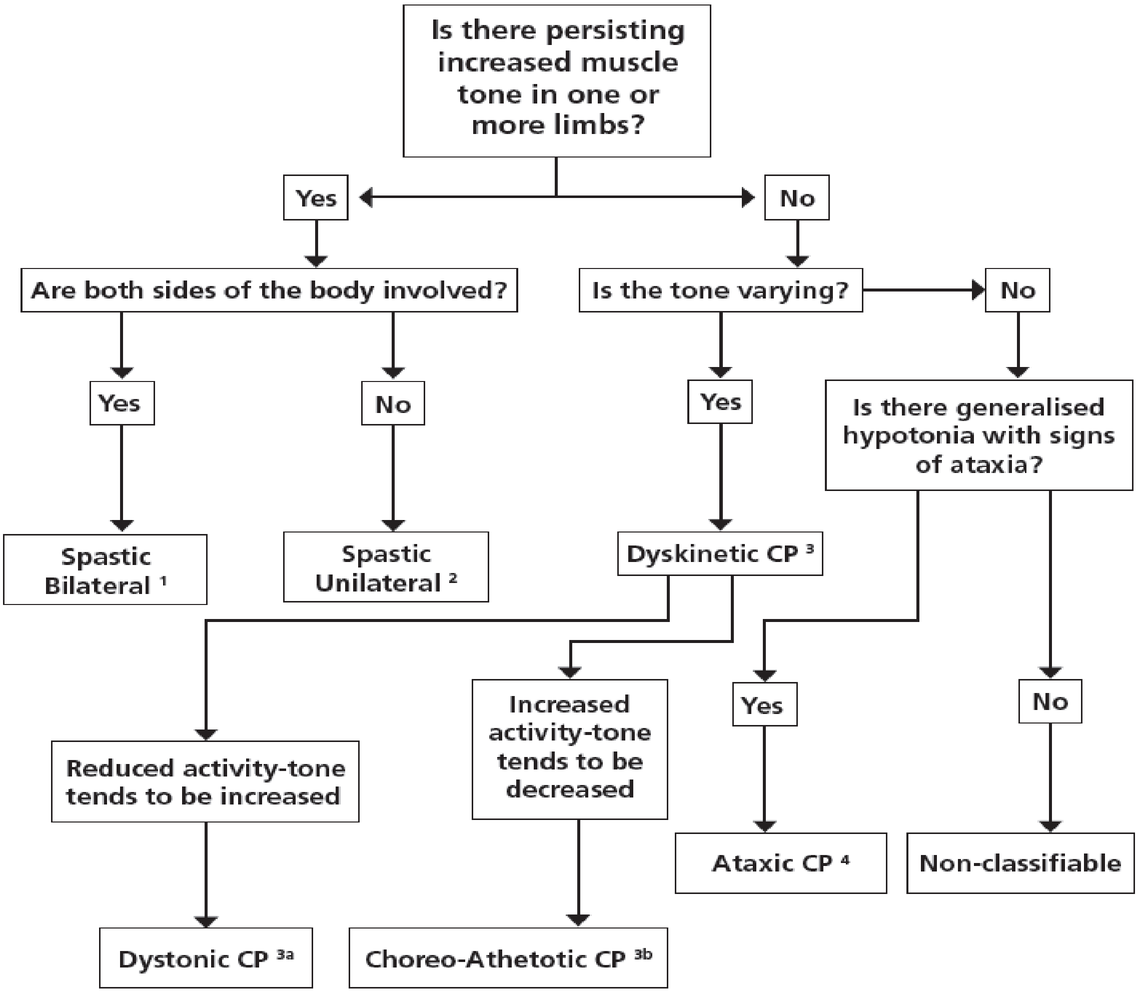

Tick the appropriate box:

Spastic, bilateral:	2 limb involvement	☐
	3 limb involvement	☐
	4 limb involvement	☐
Spastic, unilateral (hemiplegia):	Right sided	☐
	Left sided	☐
Dyskinetic:	Dystonic	☐
	Choreo-athetotic	☐
Ataxic		☐
Unclassifiable		☐

I. If **NO** , what are the alternative diagnoses? (Autism spectrum, Intellectual disability, ADHD, Hydrocephalus, Myelo-meningocele, Probable Genetic syndrome, Dysmeli, Poliomyelitis, Deafness, Speech and Language delay, etc)

____

____

____

____

Thank you

Gross Motor Function Classification System – Expanded and Revised (GMFCS – E & R)

BEFORE 2ND BIRTHDAY

LEVEL I:

- Move in and out of sitting.
- Floor sit with both hands free to manipulate objects
- Crawl on hands and knees, pull to stand and take steps holding on to furniture

- Walk between 18 months and 2 years of age without the need for any assistive mobility device.

LEVEL II:

- Maintain floor sitting but may need to use their hands for support to maintain balance
- Creep on their stomach or crawl on hands and knees.
- Infants may pull to stand and take steps holding on to furniture.

LEVEL III:

- Maintain floor sitting when the low back is supported
- Roll and creep forward on their stomachs.

LEVEL IV:

- Have head control but trunk support is required for floor sitting
- Can roll to supine and may roll to prone.

LEVEL V:

- Physical impairments limit voluntary control of movement
- Unable to maintain antigravity head and trunk postures in prone and sitting
- Require adult assistance to roll.

BETWEEN 2ND AND 4TH BIRTHDAY

LEVEL I:

- Floor sit with both hands free to manipulate objects.
- Movements in and out of floor sitting and standing are performed without adult assistance.
- Walk as the preferred method of mobility without the need for any assistive mobility device.

LEVEL II:

- Floor sit but may have difficulty with balance when both hands are free to manipulate objects.
- Movements in and out of sitting are performed without adult assistance.
- Pull to stand on a stable surface.
- Crawl on hands and knees with a reciprocal pattern, cruise holding onto furniture and walk using an assistive mobility device as preferred methods of mobility.

LEVEL III:

- Maintain floor sitting often by "W-sitting" (sitting between flexed and internally rotated hips and knees) and may require adult assistance to assume sitting.
- Creep on their stomach or crawl on hands and knees (often without reciprocal leg movements) as their primary methods of self-mobility.
- May pull to stand on a stable surface and cruise short distances.
- May walk short distances indoors using a hand-held mobility device (walker) and adult assistance for steering and turning.

LEVEL IV:

- Floor sit when placed, but are unable to maintain alignment and balance without use of their hands for support.
- Frequently require adaptive equipment for sitting and standing.
- Self-mobility for short distances (within a room) is achieved through rolling, creeping on stomach, or crawling on hands and knees without reciprocal leg movement.

LEVEL V:

- Physical impairments restrict voluntary control of movement and the ability to maintain antigravity head and trunk postures.
- Have no means of independent movement and are transported

BETWEEN 4TH AND 6TH BIRTHDAY

LEVEL I:

- Get into and out of, and sit in, a chair without the need for hand support.
- Walk indoors and outdoors, and climb stairs.
- Emerging ability to run and jump.

LEVEL II:

- Sit in a chair with both hands free to manipulate objects.
- Move from the floor to standing and from chair sitting to standing but often require a stable surface to push or pull up on with their arms.
- Walk without the need for a handheld mobility device indoors and for short distances on level surfaces outdoors.
- Climb stairs holding onto a railing but are unable to run or jump.

LEVEL III:

- Sit on a regular chair but may require pelvic or trunk support to maximize hand function.
- Move in and out of chair sitting using a stable surface to push on or pull up with their arms.
- Walk with a hand-held mobility device on level surfaces and climb stairs with assistance from an adult.
- Frequently transported when traveling for long distances or outdoors on uneven terrain.

LEVEL IV:

- Sit on a chair but need adaptive seating for trunk control and to maximize hand function.
- Move in and out of chair sitting with assistance from an adult or a stable surface to push or pull up on with their arms.
- May at best walk short distances with a walker and adult supervision but have difficulty turning and maintaining balance on uneven surfaces.
- Children are transported in the community. Children may achieve self-mobility using a powered wheelchair.

LEVEL V:

- Physical impairments restrict voluntary control of movement and the ability to maintain antigravity head and trunk postures.
- Children have no means of independent movement and are transported.

BETWEEN 6TH AND 12TH BIRTHDAY

Level I:

- Walk at home, school, outdoors, and in the community.
- Walk up and down curbs without physical assistance and stairs without the use of a railing but speed, balance, and coordination are limited.
- Participate in physical activities and sports depending on personal choices and environmental factors.

Level II:

- Walk in most settings but may experience difficulty walking long distances and balancing on uneven terrain, inclines, in crowded areas, confined spaces or when carrying objects.

- Walk up and down stairs holding onto a railing or with physical assistance
- Children have at best only minimal ability to perform gross motor skills such as running and jumping hence necessitate adaptations to enable participation in physical activities and sports.

Level III:

- Walk using a hand-held mobility device in most indoor settings.
- When seated, children may require a seat belt for pelvic alignment and balance.
- Sit-to-stand and floor-to-stand transfers require physical assistance of a person or support surface.
- When traveling long distances, children use some form of wheeled mobility. Children may walk up and down stairs holding onto a railing with supervision or physical assistance.
- Limitations in walking may necessitate adaptations to enable participation in physical activities and sports including self-propelling a manual wheelchair or powered mobility.

Level IV:

- Children use methods of mobility that require physical assistance or powered mobility in most settings.
- At home, children use floor mobility (roll, creep, or crawl), walk short distances with physical assistance, or use powered mobility.

Level V:

- Children are transported in a manual wheelchair in all settings.
- Children are limited in their ability to maintain antigravity head and trunk postures and control arm and leg movements.

BETWEEN 12TH AND 18TH BIRTHDAY

Level I:

- Youth walk at home, school, outdoors, and in the community.
- Able to walk up and down curbs without physical assistance and stairs without the use of a railing.
- Perform gross motor skills such as running and jumping but speed, balance, and coordination are limited.

- Participate in physical activities and sports depending on personal choices and environmental factors.

Level II:

- Walk in most settings.
- Environmental factors (such as uneven terrain, inclines, long distances, time demands, weather, and peer acceptability) and personal preference influence mobility choices.
- At school or work, youth may walk using a handheld mobility device for safety and may use wheeled mobility when traveling long distances.
- Walk up and down stairs holding a railing or with physical assistance
- Limitations in performance of gross motor skills may necessitate adaptations to enable participation in physical activities and sports.

Level III:

- Capable of walking using a hand-held mobility device.
- When seated, youth may require a seat belt for pelvic alignment and balance.
- Sit-to-stand and floor-to-stand transfers require physical assistance from a person or support surface.
- At school, youth may self-propel a manual wheelchair or use powered mobility.
- Walk up and down stairs holding onto a railing with supervision or physical assistance.
- Limitations in walking may necessitate adaptations to enable participation in physical activities and sports including self-propelling a manual wheelchair or powered mobility.

Level IV:

- Wheeled mobility in most settings.
- Require adaptive seating for pelvic and trunk control.
- Physical assistance from 1 or 2 persons is required for transfers.
- Youth may support weight with their legs to assist with standing transfers.
- Indoors, youth may walk short distances with physical assistance, use wheeled mobility, or, when positioned, use a body support walker.

.**Level V:**

- Youth are transported in a manual wheelchair in all settings.
- Limited in their ability to maintain antigravity head and trunk postures and control arm and leg movements

MACS; Manual Ability Classification System for children with cerebral palsy

Level I. Handles objects easily and successfully

Level II. Handles most objects, but with somewhat reduced quality and/or speed of achievement.

Level III. Handles objects with difficulty.

Level IV. Handles a limited selection of easily managed objects in simple actions.

Level V. Does not handle objects and has severely limited
